# Spatial Dependencies between Large-Scale Brain Networks

**DOI:** 10.1371/journal.pone.0098500

**Published:** 2014-06-02

**Authors:** Robert Leech, Gregory Scott, Robin Carhart-Harris, Federico Turkheimer, Simon D. Taylor-Robinson, David J. Sharp

**Affiliations:** 1 Division of Brain Sciences, Hammersmith Hospital, Imperial College London, London, United Kingdom; 2 Department of Neuroimaging, Institute of Psychiatry, King's College London, London, United Kingdom; 3 Department of Medicine, St Mary's Hospital Campus, Imperial College London, London, United Kingdom; University of Namur, Belgium

## Abstract

Functional neuroimaging reveals both increases (task-positive) and decreases (task-negative) in neural activation with many tasks. Many studies show a *temporal* relationship between task positive and task negative networks that is important for efficient cognitive functioning. Here we provide evidence for a *spatial* relationship between task positive and negative networks. There are strong spatial similarities between many reported task negative brain networks, termed the default mode network, which is typically assumed to be a spatially fixed network. However, this is not the case. The spatial structure of the DMN varies depending on what specific task is being performed. We test whether there is a fundamental *spatial* relationship between task positive and negative networks. Specifically, we hypothesize that the distance between task positive and negative voxels is consistent despite different spatial patterns of activation and deactivation evoked by different cognitive tasks. We show significantly reduced variability in the distance between within-condition task positive and task negative voxels than across-condition distances for four different sensory, motor and cognitive tasks - implying that deactivation patterns are spatially dependent on activation patterns (and *vice versa*), and that both are modulated by specific task demands. We also show a similar relationship between positively and negatively correlated networks from a third ‘rest’ dataset, in the absence of a specific task. We propose that this spatial relationship may be the macroscopic analogue of microscopic neuronal organization reported in sensory cortical systems, and that this organization may reflect homeostatic plasticity necessary for efficient brain function.

## Introduction

Functional neuroimaging studies often show increases and decreases in regional metabolism and blood flow. The pattern of increases in brain activity is highly variable, depending on the specific task demands. In contrast, decreases in activation appear superficially to have a similar spatial pattern, regardless of the nature of the task. Relative deactivation is often observed in the lateral and medial inferior parietal lobes and the ventromedial prefrontal cortex. Collectively these regions are termed the default mode network (DMN) [1], [2]. A similar spatial distribution has been observed in data acquired in the absence of any explicit task [3] and in homologous regions from resting data in non-human primate species [4] and in rodents [5].

We use the terms task positive (TP) and task negative (TN) to mean the specific pattern of relative activation and relative deactivation evoked by a specific task, without restricting this to any canonical functional brain networks (e.g., TN does not necessarily correspond to the DMN). The existence of a temporal relationship (often, seen as an anti-correlation) between some TP and TN networks is thought to be important for efficient cognitive functioning [6], [7]. For example, many cognitive tasks that require an external focus evoke increases in activity within fronto-parietal networks associated with cognitive control, which are tightly coupled with decreases in activity within the DMN [8], [9]. Although deactivation roughly consistent with a canonical DMN is reported for many different tasks, the precise pattern of regional deactivation varies with the specific task requirements [9]–[11]. This indicates that there is no fixed task negative spatial distribution, but that instead this may be malleable, depending on task demands.

**Figure 1:**
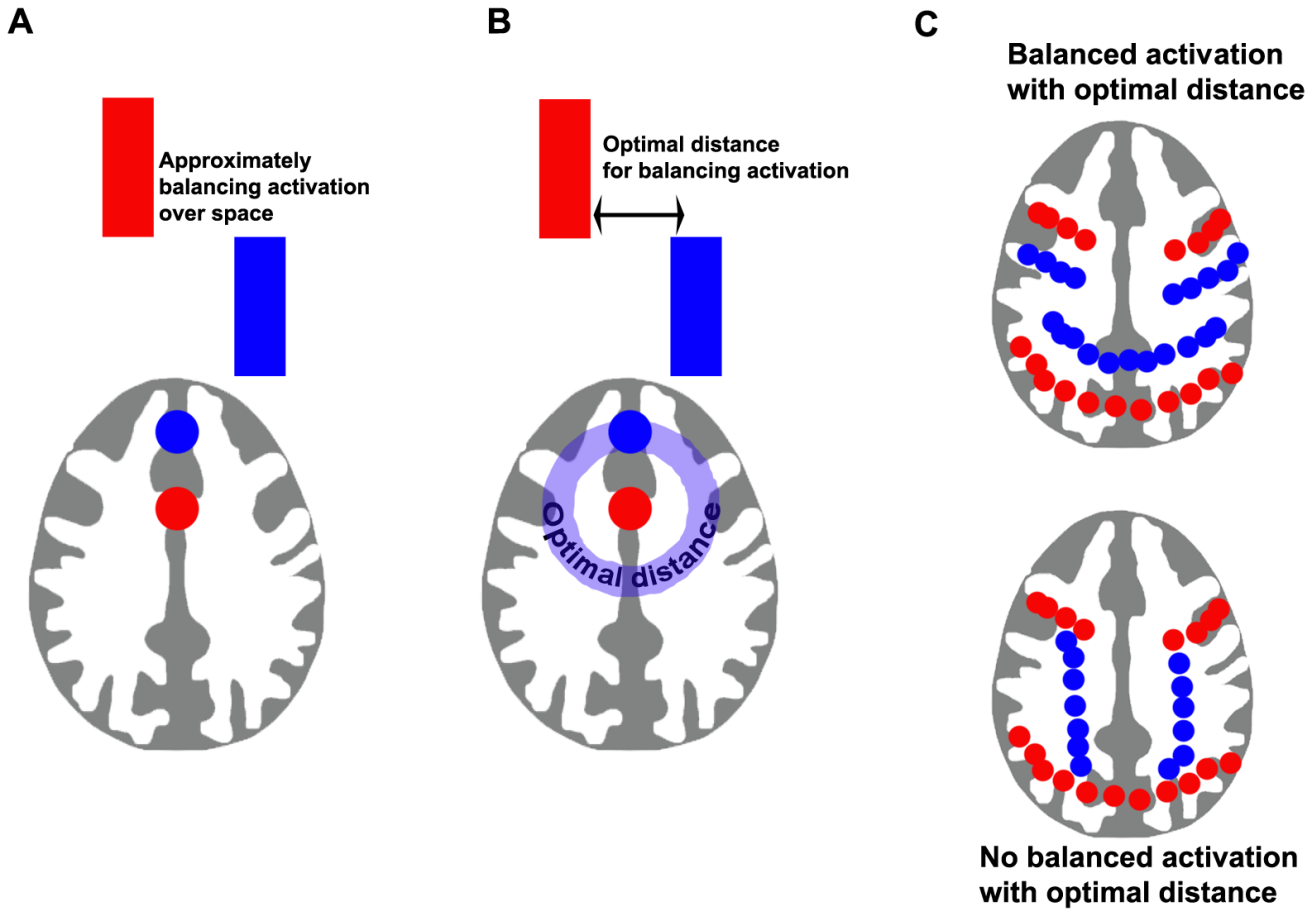
Rationale for experiment. We suggest that, in part, the spatial distribution of task negative networks may reflect underlying homeostatic processes in addition to cognitive factors. We predict that: (A) activation evoked by a task requires deactivation elsewhere in the brain; further, (B) this deactivation will occur at some optimal distance from the location of activation; therefore, (C) this pattern of deactivation will, to some degree, be “molded” to the pattern of activation, with a consistent distance between task positive and task negative voxels (top), rather than a relationship where distances are not consistent but are both long and short (bottom).

The spatial structure of the task negative network is not fixed but it is not clear why this would be. It could be as a result of a given task requiring disengagement of different neural regions depending on the cognitive requirements of the task (which is likely to be an important part of the explanation). However, in addition, it could reflect some other ongoing homeostatic process, whereby activation in a given region may involve deactivation elsewhere in the brain (Figure 1A). Such homeostatic processes are frequent in biological systems. In FMRI data, these putative homeostatic mechanisms could reflect vascular, metabolic or electrophysiological processes. If there are homeostatic processes involved in patterns of activation and deactivation with task, then there may be a spatial component to this with an optimal spatial relationship between activated and deactivated regions (Figure 1B). If there is an optimal distance for balancing activation, then this should manifest itself in TN patterns of activation that are “molded” to TP patterns (Figure 1C), such that TN voxels are located at this optimal distance from the TN voxels. Further, this specific spatial dependency between TN and TP should be relatively conserved across tasks, such that if the TP and TN voxels were from different tasks, the spatial dependency should be affected (Figure 1C).

It is important to note that we are not proposing that a homeostatic process is enough to account for the whole TN pattern observed in FMRI studies. We believe that cognitive factors will play an important role and that the TN pattern will reflect this. We also note that a homeostatic process does not require perfect balancing between TP and TN networks (i.e., exactly equal amount of deactivation to activation) depending on the specific homeostatic mechanism and its function. These factors mean that observing different numbers of evoked TN voxels versus evoked TP voxels for a given task is still consistent with some form of homeostatic process.

**Figure 2:**
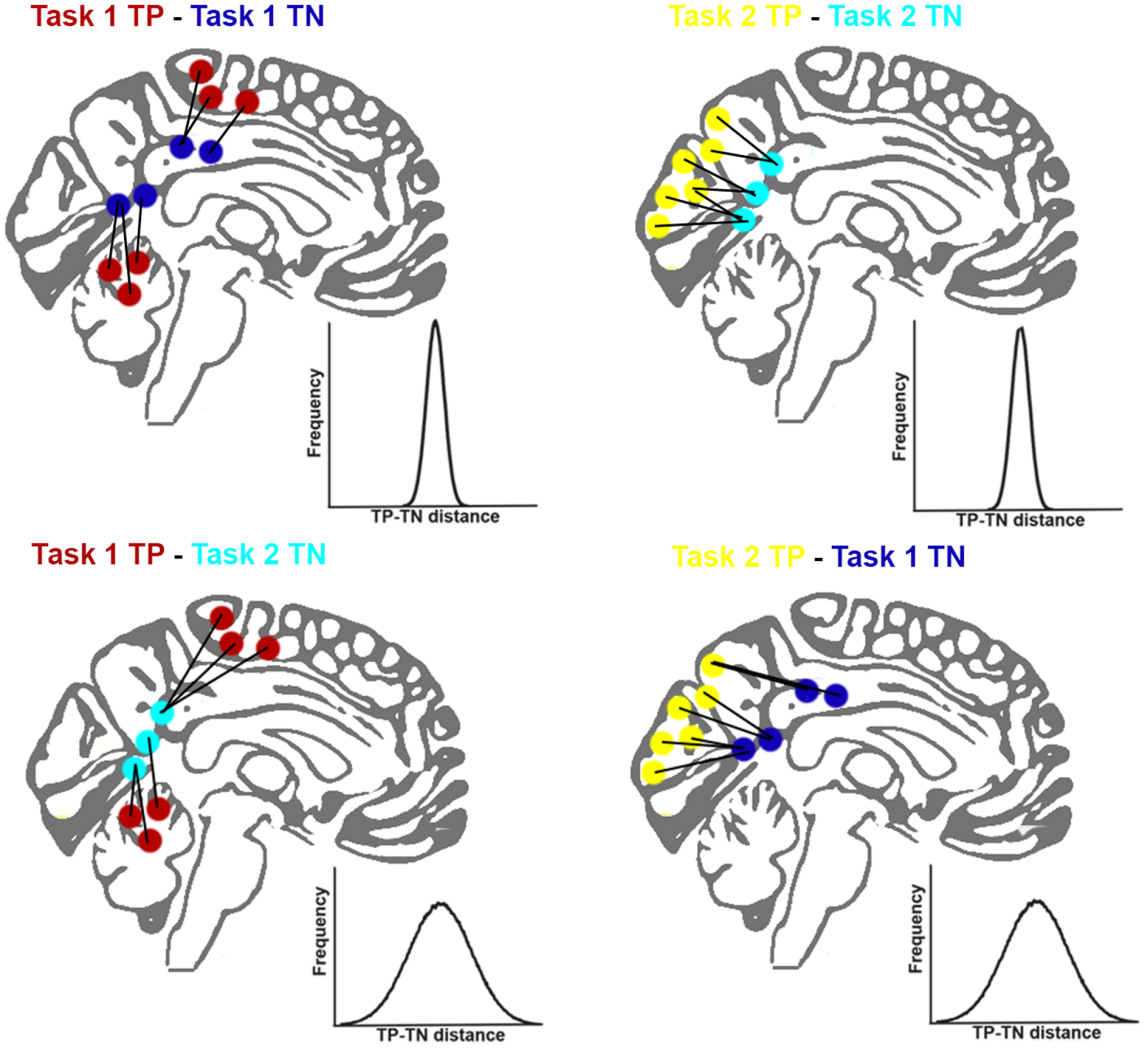
Schematic showing task positive (TP) and task negative activations (TN). The illustration schematically shows how the minimum distance between TP and TN voxels (small colored circles) was calculated on two hypothetical tasks. This is done for the TP and TN voxels from the same task and for TP voxels from one task and TN voxels from the other task. If there is no consistent spatial consistent relationship between the TP and TN networks that is sensitive to task demands, then we would expect identical distributions (i.e., represented by the width and height of the distribution) in each of the four cases. The schematic shows the experimental hypothesis that there is a spatial relationship, i.e., variance in TP-TN distance increases between different-tasks (top versus bottom) relative to within the same task (left versus right), indicating a consistent spatial dependency.

To test the hypothesis that there is a consistent spatial dependency between TP and TN patterns that may reflect some form of underlying homeostatic process, we performed an analysis of the minimal distances between TP and TN voxels evoked by different task conditions. An overview of the analytical method as applied to FMRI whole-brain data is presented in Figure 2. We predicted that if there is a consistent spatial dependency between TP and TN networks, then, for a given task, the distance between TP and TN voxels should be relatively preserved i.e., there would be a lower variability in distance between TP and TN voxels (top row, Figure 2) than would be expected if the TP and TN were taken from different tasks (bottom row, Figure 2). Further, if the spatial relationship between positive and negative networks reflects a general phenomenon of macroscopic neural organization, then it should be apparent in the absence of any task, as fluctuations in a network that occur without a task should also have spatially dependent anti-correlated networks. Therefore, we also investigated whether there is a spatial relationship between TP and TN voxels from resting data, acquired without an explicit task.

## Methods

### Participants

For Datasets 1, 2 and 3, all participants gave written consent, were checked for contraindications to MRI scanning and had no history of significant neurological or psychiatric illness. The Hammersmith, Queen Charlotte's and Chelsea research ethics committee awarded ethical approval for the study. Dataset 1 involved 21 neurologically healthy adult subjects (mean (SD) age 44 (9) years, 11 female). These subjects were scanned while alternating between viewing a static chequerboard, performing a simple finger tapping task or the rest condition with no explicit task. The raw data from Dataset 1 has been previously published in a completely different analysis and context [12]. Dataset 2 involved 18 neurologically healthy adult subjects (mean (SD) age 29 (6), 9 female) who performed a working memory task and a cued autobiographical memory task. Dataset 3 involved 68 neurologically healthy adult subjects (age range approximately between 22–35 and approximately equal numbers of males and females) who were scanned as part of the Human Connectome Project (http://www.humanconnectome.org) [13], following approval by appropriate institutional review boards in the University of Washington at St Louis and the University of Minnesota.

### MR Imaging acquisition and analysis

For Datasets 1 and 2, images were acquired using a 3 Tesla MR scanner (Intera™, Philips Medical Systems, Best, Netherlands) with an 8-channel head coil. A high-resolution T_1_-weighted sequence was acquired for all subjects.

#### Dataset 1

A single run was acquired while subjects performed simple visual or motor tasks. T_2_*-weighted gradient echo-planar images (EPI) were collected with whole-brain coverage, with the following parameters: TR = 3 s; TE = 30 ms; alpha = 90°, 2.2×2.2 axial slices, slice thickness 2.75 mm. Quadratic shim gradients were applied to correct magnetic field inhomogeneity. Subjects observed a flashing chequerboard or tapped their right or left finger on their thigh. The experiment used a blocked design with visual stimulation, finger tapping or a baseline rest condition, alternating in blocks of 24 s. There were 150 acquisitions, lasting 7.5 min.

#### Dataset 2

Data were acquired while subjects performed two separate runs: i) a 2-back working memory task; ii) an autobiographical memory task. T_2_*-weighted gradient echo planar images were collected with whole-brain coverage, with the following parameters: repetition time, 2 s; echo time, 30 ms; alpha = 90°; 31 slices; slice thickness 3.25 mm; interslice gap of 0.75 mm; acquisition in ascending order (resolution: 2.19, 2.19, 4.0 mm). Quadratic shim gradients were used to correct for magnetic field inhomogeneities within the brain. The 2-back working memory task was a blocked design. There were five 36 second blocks of task, interspersed with 10 second rest blocks. During the task, subjects saw single pictures presented sequentially on the screen. Stimuli were presented for 1.75 s with an inter-stimulus interval of 0.5 s (16 stimuli per block). Target stimuli occurred in 20% of trials. Subjects responded with a finger press when the picture was the same as that presented two pictures previously. Subjects were instructed to respond as quickly and as accurately as possible. The autobiographical memory task involved cued retrieval of personally relevant memories. Preparation for the involved subjects providing a list of written memory cues (e.g., “Remember the engagement party”) at least two days prior to scanning. In the scanner, these cues were visually presented and the subjects were asked to think about the autobiographical memory cued. As with the 2-back task, five 36 second blocks of the autobiographical memory task were interspersed with 10 second rest blocks. The written prompts for the subject's memories were presented for 17.5 seconds with an inter-stimulus interval of 0.5 s (2 stimuli per block).

#### Dataset 3

This data was collected as part of the Human Connectome Project. It was composed of 1400 gradient-echo echo planar images, collected with whole-brain coverage, with the following parameters: repetition time = 720 ms; echo time = 33.1 ms; flip angle = 52 deg; FOV = 208×180 mm (RO×PE); Matrix = 104×90 (RO×PE); Slice thickness 2.0 mm; 72 slices with 2.0 mm isotropic voxels; Multiband factor = 8; Echo spacing = 0.58 ms; and bandwidth = 2290 Hz/Px. For each participant, two runs were acquired one with the phase encoding direction of the images from right-to-left phase encoding and the other from left-to-right.

*For Datasets 1 and 2*, whole-brain fMRI data were analyzed individually with standard general linear models analysis tools from the FSL library (FEAT version 5.98). For all runs from all subjects, image pre-processing involved realignment of EPI images to remove the effects of motion between scans, spatial smoothing using a 6 mm full-width half-maximum Gaussian kernel, pre-whitening using FMRIB's improved linear model (FILM) and temporal high-pass filtering using a cut-off frequency of 1/50 Hz to correct for baseline drifts in the signal. FMRIB's Linear Image Registration Tool was used to register EPI functional datasets into standard MNI space using the participant's individual high-resolution anatomic images. FMRI data of each individual subject were analyzed using voxel-wise time series analysis within the framework of the General Linear Model (GLM). To this end, a design matrix was generated with a synthetic hemodynamic response function and its first temporal derivative. The timecourses from motion parameters were also included to attempt to account for noise related to head motion. For dataset 1, timecourses of visual stimulation and left and right finger tapping were modeled in the design matrix for each subject, with rest as an implicit baseline. For dataset 2, each run was analyzed separately, with a simple design matrix modeling the blocks when tasks were being performed (either 2-back or autobiographical memory task) versus the implicit rest baseline.

T-statistic images for each individual participant, for each task were calculated. These statistical maps were then thresholded (both positive to derive task positive maps and negative values for task negative maps), binarized, resampled into 4 mm×4 mm×4 mm voxel resolution and then used to assess the spatial relationship between TP and TN (see below) voxels.

To illustrate the approximate distributions of TP and TN voxels used in the spatial variability analyses, group maps were created from the individual t-stat images. Each subject's t-stat maps was thresholded at t>3, binarized and subsequently the mean across subjects was calculated, to create a proportion overlap map. The proportion overlap maps for TP and TN voxels are presented below. Voxelwise higher-level group GLM results, corrected for multiple comparisons are not presented, since our hypothesis does not involve asking whether any specific voxel is active at the group level or not.

*For Dataset 3*, the data was preprocessed as part of the Human Connectome Project [14]. In brief, this included: (i) correction for field inhomogeneities, (ii) motion correction of the data; (iii) transfomation and resampling into 2 mm×2 mm×2 mm MNI152 standard space. In addition, the data: (i) was high-pass filtered using a 100 second filter; (ii) spatially smoothed with a 5 mm FWHM Gaussian kernel; (iii) downsampled into 4 mm×4 mm×4 mm (this was done for computational reasons, given the size of the datasets). Subsequently, an independent component analysis (ICA, using Melodic 3.13) was conducted on each individual's data, extracting 70 components. To derive whole-brain group networks, the 70 components from each subject were entered into a temporal concatenation group ICA, extracting 20 components. This hierarchical approach (running individual ICA before concatenating the results and running a higher-order ICA) was done because of the large size of the dataset (1400 whole brain images for 68 subjects). Non-noise components from the group-level analysis were then selected and used to assess spatial relationship.

### Assessing the spatial relationship between TP and TN

Figure 2 illustrates the general approach to measuring the spatial relationship between the TP and TN. To do this, we estimated the minimum Euclidean distance in number of voxels from each TP voxel to the nearest TN voxel. This resulted in a distribution of distances that was then used to calculate the variability of distances, for each subject for each experiment. Two measures of variation of minimum distances (the Gini coefficient and the coefficient of variation) were calculated as well as the mean minimum distance for each subject. The Gini coefficient is a standardized measure of the equality of a frequency distribution (with a value of 0 meaning that there is no variability and a value of 1 meaning maximal variability) and the coefficient of variation is simply the standard deviation scaled by the mean distance. Both are standardized measures of the variability of a distribution that are unaffected by the mean value of the distribution. Standardization was done because there are different numbers of voxels activated or deactivated in different tasks (e.g., because of differences in the sensitivity of the tasks, regional differences in neural signal and non-neural noise sources etc). These different numbers of voxels could alter the mean distances between the TP and TN, and as a result affect non-standardized measures of variability. Below, we report results with the Gini coefficient; however, the coefficient of variation provided qualitatively the same pattern of results.

There were two tasks within both Datasets 1 and 2, resulting in four possible TP to TN distributions of minimal distance for each dataset (task 1 TP to TN; task 1 TP to task 2 TN; task 2 TP to task 2 TN; and task 2 TP to task 1 TN: see Figure 2). Variability measures based on these distributions were subsequently compared in a general linear model, with within/across-task and task-type as repeated measures factors. Under the null hypothesis, if there was no spatial relationship between TP-TN voxels, then the variability would be the same if the TP and TN voxels were taken from the same task or from different tasks. Therefore, we were primarily interested in the comparison of variability of within versus across tasks. Using real patterns of TP and TN voxels from different tasks to formulate the null hypothesis is preferable to using randomly generated data (e.g., comparing the TP pattern of activation with randomly placed TN voxels). This is primarily because it is not clear how to randomly place TN voxels in a way which is biologically plausible: in the real brain voxels are not spatially independent both because of the spatial extent of neural activity (extending across multiple neighboring voxels) as well as non-neural constraints such as vasculature and non-neural noise sources which are shared by nearby voxels. Therefore, comparison of real versus random placements of voxels does not form a simple null hypothesis to test for the presence of a TP-TN spatial relationship. Equally, using spatial overlap (e.g., correlation or mutual information) between t-statistic maps to assess spatial dependency is not appropriate since the specific question addresses whether there is a consistent spatial dependency between non-overlapping TP and TN networks that is persists across tasks.

For a given t-threshold, the minimum distribution of distances was calculated separately for each participant and for each task; statistics summarizing the distribution of distances were taken to a higher-level for comparison across participants. For a given dataset, the same t-threshold was applied to every subject's data, to both tasks, and for TP and TN maps equally. No explicit statistical comparisons were made between dataset 1 and dataset 2, where participants, MR acquisition parameters, and task design parameters such as length of task blocks, differed. Instead all analyses were constrained to within a dataset, where everything (task block length, number of blocks, participants, acquisition parameters) apart from the cognitive and perceptual task demands were held constant across tasks and so could not explain differences in the distribution of TP and TN voxels. To show the robustness of results, the data was thresholded at a range of t-values in the range t = 1.7 (nominally approximately p = 0.05) to 3 (p<0.005; at t thresholds greater than 3, increasing numbers of subjects had no voxels activated for either the TP or TN in at least one task). Qualitatively similar results were found across the range of t-values; therefore, we present results at voxel thresholds of t = 2 and t = 3. Correcting the voxelwise statistics for multiple comparisons was not appropriate at this stage because the hypothesis was not about the presence of voxel specific activation, but rather the brain-wide distribution of TP to TN distances.

Non-neural phenomena such as vascular stealing have been hypothesized to result in relative decreases in activation that are spatially adjacent to areas of increased activity [15]. If vascular stealing does occur then the TN voxels can be assumed to be of two types: (i) vascular stealing TN voxels that are spatially near to the TP voxels and (ii) other TN voxels (e.g., putative DMN voxels) that are not theoretically spatially near to the TP voxels. Type (i) TN voxels would be more frequent in within-task comparisons of TP-TN minimal distances than across-task comparisons and would have short minimal distances. Whereas type (ii) TN voxels would (under the null hypothesis) be equally likely for both within and across task analyses. Therefore, if type (i) TN voxels exist, they would make the mean minimal distance lower for the within task analyses than the across task analyses. They would also result in a difference in the skewness (towards short distances) for the within-task analyses. This logic also applies to any other phenomena that might lead to nearby decreases in activation, e.g., surround inhibition. To ensure that differences between within and across-task spatial variability were not dependent on this type of artifact, we calculated the mean and the skewness of each frequency distribution of distances. As a further check, we also repeated the spatial variability analyses, excluding all TP to TN distances at or below a minimum Euclidean distance of two voxels (8 mm). The minimum distance analysis was performed to test if the results are consistent even without nearby voxels, which are more likely to share vasculature. Results below are presented both including all TP to TN distances and excluding Euclidean distances less than two voxels.

### Assessing the spatial relationship at rest

For Dataset 3, the general procedure was similar; however, instead of using individual subjects' data, group maps were used. Each non-noise component was thresholded using either z = 2, z = 3 or z = 4 and binarized, resulting in positively and negatively coupled networks for each component. The minimum Euclidean distance from positive to negative voxels was calculated for each positive voxel, and a distribution of distances and measures of variability (i.e., Gini-coefficient) calculated. Just as with the task data, under the null hypothesis that there is no spatial relationship between positively and negatively-coupled networks, then assessing distance from positive and negative networks from different components should result in the same variability as from when both positive and negative networks are from the same component. To test this, variability in distances for voxels from positive and negative networks within a component were compared to variability for networks from across components.

## Results

### Task positive and task negative regions at the group level

**Figure 3:**
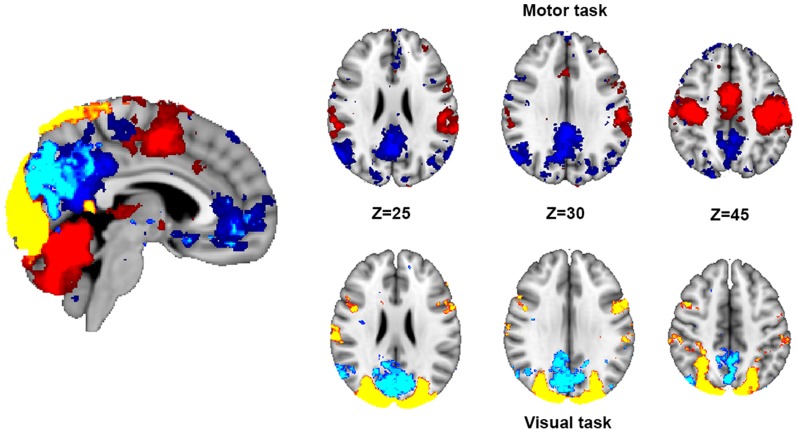
Motor and visual task activation. Voxels that are activated (t-threshold>2 for >20% of subjects) for the motor condition are in red (task positive: task > rest) and voxels deactivated are in dark blue (task negative: rest > task) colors. The visual condition is in yellow (task positive) and light blue (task negative). We note that the both conditions are from the same run in the same participants with the same acquisition parameters (i.e., block length, number of TRs, length of TR, echo time etc). Therefore, the difference in the spatial distribution between the two TN networks is unlikely to be because of differences in statistical power between the conditions.

The pattern of relative activation (TP) and deactivation (TN) for two tasks (a visual checker board or a finger tapping task) was analyzed across subjects (Figure 3). This showed the expected pattern of activation in visual occipital regions (lighter warm colors) for the visual task and sensorimotor parietal, frontal and cerebellar regions for the motor task (darker warm colors). Whereas the tasks evoked highly distinct spatial patterns for relative activation (TP), the patterns of relative deactivation (TN) were more similar across the two tasks, with some overlapping regions within the lateral parietal and occipital regions. However, despite this broad similarity, the visual (light blue) and motor (dark blue) tasks evoked different patterns of deactivation, with the visual TN voxels being generally more posterior. Therefore, the group data demonstrate distinct patterns of activation and deactivation, i.e. spatially different TP and TN networks.

### The distance between task positive and task negative voxels is less variable within a task than across tasks

**Figure 4:**
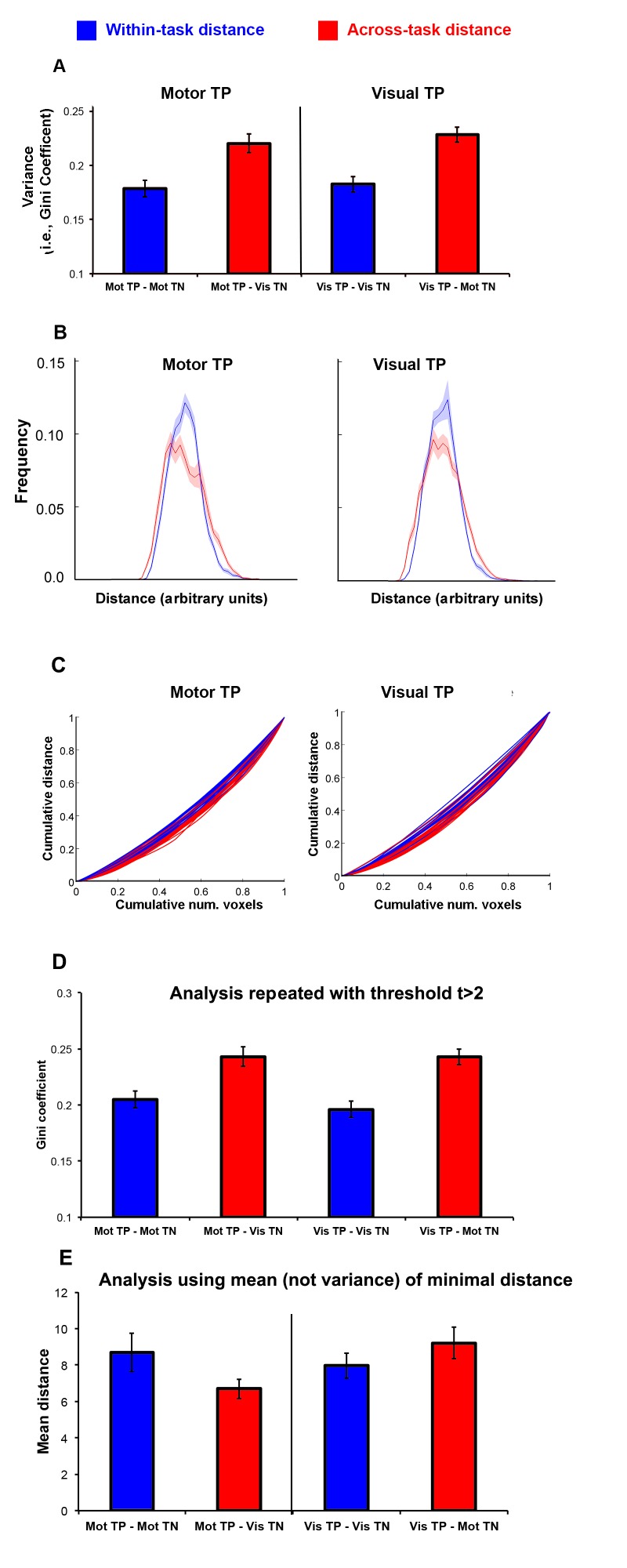
Variability in distance for motor and visual tasks. Variability in the distance between task positive and task negative (measured by the Gini coefficient) voxels for the motor and visual tasks, comparing intra-task versus inter-tasks. 4A: the average Gini coefficient using a t-threshold >3, before calculating distance. 4B: the normalized frequency distribution of minimum TP-TN distances for both within task (in blue) and across task (in red). For each individual, the distribution of distances was divided by the mean and centered on zero to normalize it to allow calculation of the group mean frequency distribution (solid lines) and the standard error (shaded areas) above and below the mean. 4C: individual subjects' Lorenz curves (a graphical representation of the Gini coefficient). For each subject, the distances from smallest to largest are plotted against the cumulative frequency: blue is within task, red is TP-TN distance between tasks (the closer the line is to the 45^o^ line, the lower the variability). The intra-task curves (blue lines) are on average closer to the 45^o^ line than the inter-task curves (red lines) for both visual and motor task positive voxels; 4D: the analysis repeated using a more liberal threshold of t>2; 4E: the mean TP-TN minimum distance for within (blue) and across task (red) with standard error bars.

To assess whether there is task-dependent modulation of the spatial relationship between the TP and TN voxels, the distance between TP and TN voxels was calculated and the variability of these distances was then compared within a task and across different tasks. Figure 4 shows the variability in the distances (as measured by the Gini coefficient) between visual and motor TP voxels and TN voxels - either across or within tasks, both at the group level (Figure 4A; 4B: plotting normalized frequency distributions of distances for across and within tasks) and for individuals (Figure 4C: plotting Lorenz curves for each individual's distribution of distances for within and between subject, the closer the line is to the 45^o^ line, the less variability for that individual). A repeated measures general linear model showed that within-task variability was significantly lower than across-task variability (F(1,20) = 59.8, p<0.001), consistent with a task-dependent modulation of the TP-TN network spatial relationship. Similar results were also obtained using non-parametric approaches (e.g., Wilcoxon signed-rank tests). This result was observed using a threshold of t>3 to define TP and TN voxels, and the same pattern of results was observed: when the threshold was t>2 (F(1,20) = 67.7, p<0.001). Qualitatively similar results were found using a different measure of variability (the coefficient of variability), also at t>3 and t>2 (see Figure 4D). The reduced variability in distance within task could not be explained by differences in the mean minimum distance between TP and TN voxels (Figure 4E), where mean distances were not significantly different in the within versus across task comparison, or between the two tasks.

It is possible that non-neural factors could affect the distance distribution differently for within than across task. One possible confound is that it is logically possible for TP and TN from different tasks to overlap, but not within the same task. It is also theoretically possible that the underlying vasculature might influence the results. For example, a “vascular steal” between voxels with shared vascular was proposed as an explanation of negative BOLD effects [16] (although many studies show that negative BOLD is neural not vascular in origin e.g., [17]). Either of these possibilities would theoretically introduce an artefactual skew in the distribution. To rule out these possibilities, we examined the skewness of the distances as well as their variability. We found no significant effect of within- versus across-task (F(1,20) = 0.029, ns) on skewness. As a further demonstration that the result are not an artifact of vascular stealing, we also reran the variability analyses while removing distances that were a minimum Euclidean distance of 2 or fewer voxels apart (therefore, ensuring that nearby voxels that might share microvasculature are not included in the analysis). As with the prior analyses, even while removing small distances, there was a significant main effect of within the same condition versus across different conditions (F(1,20) = 33.9, p<0.001).

### A consistent spatial relationship between task-positive and task-negative networks are seen across cognitive tasks and individuals

**Figure 5:**
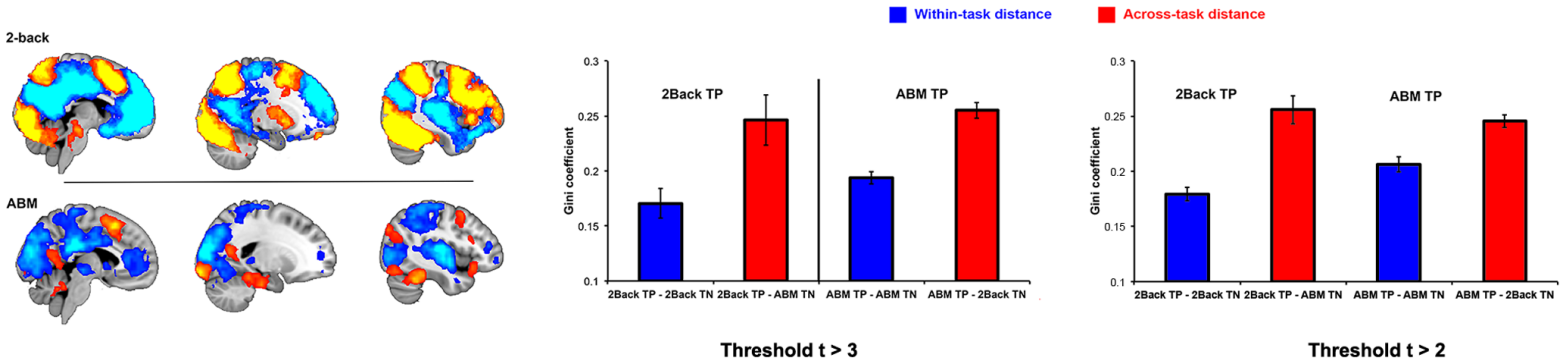
Working memory task activation. A second example using two different, cognitive tasks from a separate dataset. In this case, a 2-back working memory task and an autobiographical memory task. A: TP and TN voxels (t>2, present in >20% of subjects) are presented for the two tasks. B: variability of minimum distances between TP and TN at either a threshold of t>3 or t>2. Again, the subjects, stimulus and acquisition parameters are the same in both tasks, meaning that differences in TP and TN distributions are unlikely to be artifacts relating to statistical power.

The same pattern of results is seen in a different set of subjects performing two different tasks that evoke very different patterns of activation and deactivation: a working memory 2-back task and a cued autobiographical memory task. Figure 5A shows the pattern of TP and TN voxels across subjects. This shows some overlap with the TP regions for both tasks (e.g., in medial prefrontal regions) but also considerable spatial differences (e.g., in visual, parietal and retrosplenial regions). Similarly, the TN regions were different between the two tasks. Figure 5B shows the variability in TP-TN distances for the different tasks (both within- and across-tasks) for thresholds of t>3 and t>2. We observed the same pattern of lower within versus across task variability in TP-TN distance (thresholded at t>3: F(1,14) = 58.2, p<0.001; thresholded at t>2: F(1,17) = 59.8 p<0.001; and with a minimum distance of at least two voxels: F(1,14) = 12.8, p<0.005). As with the previous dataset, there was no evidence of a difference in skewness when comparing within versus across tasks (F(1,14) = 0.3, ns).

### The same reduction in variability is present for positive and negatively correlated networks derived without an explicit task

**Figure 6:**
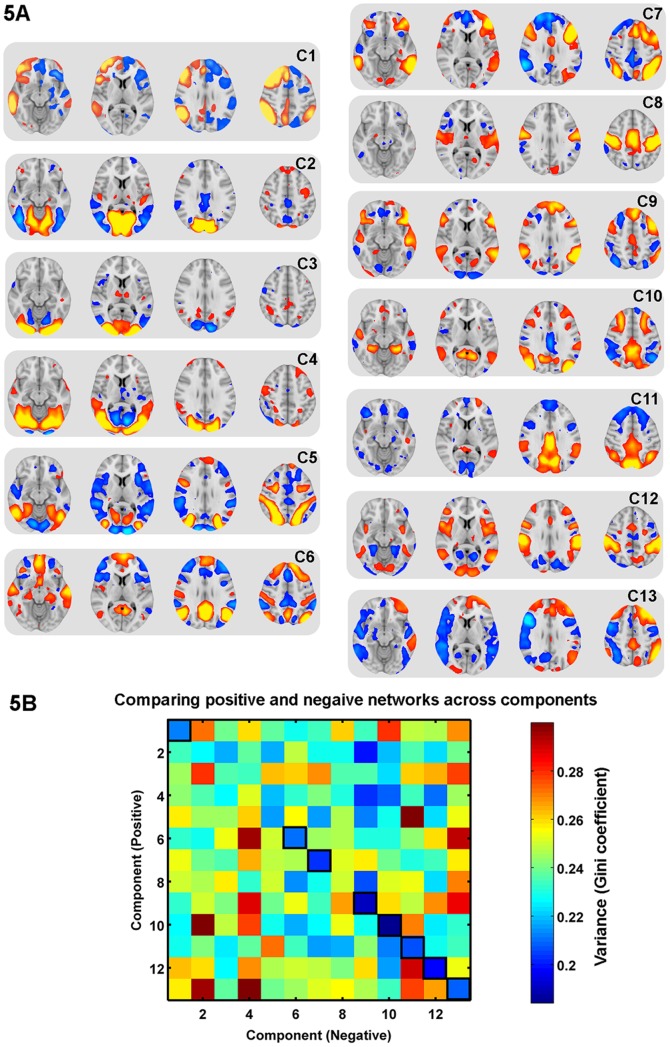
Variability of distances at rest. A: Thirteen non-noise independent components calculated from the Human Connectome Project resting state data (thresholded at z>2). Each component consists of both positively covarying and negatively covarying networks of brain regions. B: The Gini coefficient of the distribution of minimum Euclidean distances between positive and negative networks for different components; values along the diagonal are within a component, non-diagonal values are from across components.

As a final investigation of a spatial relationship, we also considered positively and negatively covarying networks defined in the absence of an explicit task i.e. a ‘resting state’ condition. An independent component analysis was run on 68 subjects' data acquired at rest as part of the Human Connectome Project. Twenty independent components were extracted: of these, seven components were judged to be noise (because they were predominantly either outside the brain or within white matter or CSF), leaving 13 putative neural components. These components are presented in Figure 6A. In all components, brain regions with significant positive and negative correlation are apparent. Each component was thresholded into both a negative and a positive network, resulting in 13 positive and 13 negative networks. The minimum Euclidean distance between positive and negative voxels was compared either within a component (e.g., positive voxels from component 1 with negative voxels from component 1) or across components (e.g., positive voxels from component 1 with negative voxels from component 2). This resulted in a distribution of minimum distances, the variability of which was assessed with the Gini-coefficient. Figure 6B shows this variability in minimum Euclidean distance between positive and negative networks from the 13 components, at the threshold z>2. This shows that the lowest variability is found along the diagonal, where the positively and negatively-coupled networks come from the same component. Eight of the 13 components had lowest variability within a component (i.e., lie on the diagonal of Figure 6B) compared to any of the other components (three of the five components that did not show lowest within component, were predominantly restricted to the occipital lobe). A t-test comparing within- versus between-components revealed significantly lower variability in minimum distances within a component (t(167) = 4.87, p<0.001), a result that was replicated at different z thresholds (z>3, t(167) = 2.68, p<0.01; z>4, t(167) = 1.99, p<0.05). These results are, therefore, in agreement with the results from the two task datasets, in suggesting a spatial relationship between positive and negative networks.

## Discussion

Across four different tasks, with very different cognitive and sensorimotor requirements, the minimum distance between task positive (TP) and task negative (TN) voxels display less variability than would be expected by chance. Similar results for positively and negatively-coupled networks are found for ‘rest’ data. These results show that although TP and TN networks are task-dependent, the spatial relationship between them is relatively preserved. Moreover, it suggests that the spatial organization of TP and TN voxels are inter-dependent. For example, a visual stimulus activating occipital regions will be accompanied by a mirroring pattern of deactivation in occipital and medial parietal regions, whereas finger tapping activating motor cortical regions will be accompanied by more dorsal and anterior deactivation. This relationship can be considered as a spatial analogue of the temporal relationship (e.g. anti-correlations) frequently reported between some task positive and task negative networks [18]–[21], and provides an important explanatory principle for the organisation of patterns of brain network activity.

There are a number of possible explanations for the existence of the spatial dependency between TP and TN networks that we have systematically demonstrated. One possibility is that due to the extremely high metabolic demands of neural activity [22], any change in regional activity must be accompanied by some amount of balanced deactivation across space. This inclination for a spatial relationship between the networks may reflect a requirement of the global system to maintain a critical level of activity, perhaps implying that functional brain systems compete for what is a finite metabolic quota. Similarly, the spatial relationship that we observe between TP and TN networks may exist in order to prevent decompensation and runaway excitation. The implication is that for any particular TP pattern, an accompanying and spatially predictable TN network is required in order to stabilize the global metabolic activity. It has been argued that the brain operates at or near a critical state [23]–[28]. This is proposed to allow optimal information processing and the rapid and efficient exploration of multiple different network states. Consistent spatial relationships are observed in critical systems across spatial scales [23], [29]. By analogy with this literature on criticality, the existence of spatial relationships between networks may allow the brain to maintain useful, persistent dynamics, without crossing into either highly stable or unstable states (analogous to sub-critical or super-critical, e.g., having epileptic seizures).

A related explanation is that the spatial relationship of competitive systems has benefits for efficient information processing. A model of balanced activity where neurons with a positive response are coupled to neurons with negative responses has been proposed to operate at the neuronal level within primary visual and auditory cortices [30]. At the neuronal level such a coupling explanation arises because neurons use an efficient coding in which redundant information is discarded [31]. Receptive fields of mammalian visual and auditory cortices are spatially organized, operate across a range of spatial scales, and can be understood as banks of Gabor (or wavelet) filters [32]–[34]. These filters remove statistical redundancy and increase independence between neuronal responses to natural stimuli. However natural sounds and images exhibit strong statistical dependencies that cannot be eliminated with linear operations alone. [30] proposed a simple and efficient solution to this problem by demonstrating that linear filter banks are optimal for coding as long as the output of each filter is rectified (divided) by a weighted sum of the spatially linked (and time/frequency conjoint) filter outputs. Therefore, positive neuronal responses to a sensory input are intrinsically coupled to negative responses. We suggest that this property, found in sensory cortex, may be present at a larger spatial scale, and describe the system-level patterns of activation observed here at the macroscopic scale. Similarly, this phenomenon may not be limited to just sensory stimuli but may describe a more general organizational principle that applies to all information processing in the brain.

An alternative and less interesting explanation is that the observed spatial pattern is a result of local changes in cerebral blood flow or volume, possibly reflecting hemodynamic processes. As blood flow increases in task positive regions, to meet local metabolic needs, neighboring regions experience compensatory reductions in blood flow (“vascular steal”), leading to local changes in BOLD [15], [16]. These haemodynamic factors are unlikely to explain our results for a number of reasons. Firstly, while vascular stealing has not been definitely ruled out as an explanation of findings of negative BOLD, a number of studies have robustly shown that negative BOLD reflects underlying neural activity [17]. Secondly, the TP and TN networks and the resting state networks do not correspond to vascular anatomy, in either their overall organization or the way they dynamically change with task context. Thirdly, we showed that there is no difference in mean distance or skewness of the distance distribution when comparing within versus across task data, and finally we observe the spatial relationship even when only considering task positive and task negative voxels that are a minimum distance (>8 mm) apart, which would eliminate much of the microvasculature proposed to explain the “vascular steal”.

We have examined the relationship between TP and TN at a single spatial scale, but a similar relationship between excitation and inhibition has been proposed to operate at the microscopic level [30]. Therefore, it is possible that the spatial relationship we describe between activation and deactivation is a feature of the critical organization of the brain. Future work should investigate how this relationship operates across scales, and test the hypothesis that the spatial inter-dependence between activation and deactivation is scale-free (scale-free temporal characteristics have been reported within both TP and TN regions [35]. Equally, further work is needed to evaluate different methods for measuring spatial differences between task-evoked patterns of activation and deactivation across different tasks. Here, we used Euclidean distances in three-dimensional MNI space since we wanted to allow for both small (intraregional) distances as well as interregional distances. An alternative to our approach would be to measure variability on distances between activation and deactivation on a two-dimensional unfolded cortical surface. The underlying structural connectivity pattern might also reflect the interaction between TP and TN networks, and diffusion imaging could be used to assess if the long-range white-matter tract organization reflects these spatial patterns. For example, future work could investigate if the graph-theoretic path-length between TP and TN regions is shorter and more consistent than expected by chance.

Whatever the explanation of the spatial dependencies, their very existence challenges some of our understanding of the functional role of task-evoked changes in activation and, in particular, the role of a canonical DMN [1] and its relationship to observed task negative networks. The canonical DMN is reported to be broadly similar across a range of externally focused tasks; the work presented here suggests that the precise boundaries of deactivation are molded in a way that reflects the pattern of task positive activation. One possibility is that the DMN can be thought of as containing a core of regions (the DMN proper with specific functional mappings onto e.g., stimulus independent thought) that consistently show relative deactivation by a range of tasks, combined with more peripheral regions that adaptively respond to the specific neural or metabolic requirements of the task. Alternatively, the DMN may not be a single coherent network at all, but instead is a convenient fiction; i.e., there is a separate TN network for each TP network, and the DMN represents a subset of all possible TN networks that have somewhat similar spatial distributions. Future work will be needed to disambiguate these two possibilities and further explore its implications for networks similar to TN networks that are observed at rest.

In summary, we provide evidence for the existence of spatial relationships between task positive and task negative networks that are consistent across tasks and individuals and exist even in the absence of an explicit task. One possible interpretation is that there is a homeostatic organization of dynamic changes in large-scale network activity, where global activity is balanced to prevent decompensation and perhaps to promote efficient neural coding.
